# Colorectal cancer after a negative Haemoccult II® test and programme sensitivity after a first round of screening: the experience of the Department of Calvados (France)

**DOI:** 10.1038/sj.bjc.6990692

**Published:** 1999-09

**Authors:** V Bouvier, G Launoy, C Herbert, H Lefevre, J Maurel, M Gignoux

**Affiliations:** Registre des Tumeurs Digestives du Calvados, CJF INSERM 96-03, Faculté de Médecine, Avenue de Côte de nacre, Caen, 14032 cedex, France; Service de Chirurgie Digestive, CHU, Avenue de Côte de nacre, Caen, 14033 cedex, France

**Keywords:** colorectal cancer, screening, false-negative test, Haemoccult II®, programme sensitivity

## Abstract

Colorectal cancers emerging after a negative Haemoccult II® are described in the context of a first round of mass screening in the Department of Calvados (France), from April 1991 to the end of December 1994. People with a cancer occurring after a negative test until 31 December 1995 were identified by a local cancer registry. Incidence was calculated and the programme sensitivity was estimated. The incidence of cancer emerging after a negative test was 57.7 per 100 000, i.e. half of the calculated incidence in the reference group (141.6 per 100 000). These cancers did not differ from those of either the non-responder or reference groups, in particular for the stage of extension. The programme sensitivity was globally higher than that estimated in European trials: 77.2, 66.3 and 55.9%, 1, 2 and 3 years after the test respectively. Programme sensitivity was higher for distal colon cancer 1 year after the test, which is probably due to the relatively slow growth of this subsite. © 1999 Cancer Research Campaign


*British Journal of Cancer* (1999) **81**(2), 305–309
© 1999 Cancer Research Campaign
Article no. bjoc.1999.0692

### Colorectal cancer after a negative Haemoccult II® test and programme sensitivity after a first round of screening: the experience of the Department of Calvados (France)

**V Bouvier[1], G Launoy[1], C Herbert[1], H Lefevre[1], J Maurel[1,2] and M Gignoux[1,2]**

[1]Registre des Tumeurs Digestives du Calvados, CJF INSERM 96-03, Faculté de Médecine, Avenue de Côte de nacre, 14032 Caen cedex, France;
[2]Service de Chirurgie Digestive, CHU, Avenue de Côte de nacre, 14033 Caen cedex, France

**Summary** Colorectal cancers emerging after a negative Haemoccult II® are described in the context of a first round of mass screening in the Department of Calvados (France), from April 1991 to the end of December 1994. People with a cancer occurring after a negative test until 31 December 1995 were identified by a local cancer registry. Incidence was calculated and the programme sensitivity was estimated. The incidence of cancer emerging after a negative test was 57.7 per 100 000, i.e. half of the calculated incidence in the reference group (141.6 per 100 000). These cancers did not differ from those of either the non-responder or reference groups, in particular for the stage of extension. The programme sensitivity was globally higher than that estimated in European trials: 77.2, 66.3 and 55.9%, 1, 2 and 3 years after the test respectively. Programme sensitivity was higher for distal colon cancer 1 year after the test, which is probably due to the relatively slow growth of this subsite.
© 1999 Cancer Research Campaign

**Keywords:** colorectal cancer; screening; false-negative test; Haemoccult II®; programme sensitivity

Colorectal cancer is frequent in Western countries. In France, with 33 400 cases per year (Benhamiche et al, 1997), it is the most frequent cancer for both sexes and represents about 15% of all malignant tumours (Faivre et al, 1997). Several mass-screening trials took place in the 1980s, essentially in Anglo-Saxon countries. Haemoccult II®, the most frequently used faecal occult blood test until now, has had its efficacy proven in three controlled trials (Mandel et al, 1993; Hardcastle et al, 1996; Kronborg et al, 1996), with a significant reduction of colorectal-specific mortality between the screened groups and the control groups.

Unfortunately, the benefit obtained is low and the extension of screening to the general population in France must overcome two problems: the poor participation rate and the relatively low sensitivity of the test. In the literature the definition of sensitivity is variable. In fact, it is important to distinguish test sensitivity and programme sensitivity. The latter, which is the most frequently used, corresponds to the ability of a screening programme to detect a cancer, and can be directly estimated with the ratio a/a + c where ‘a’ is the number of cancers detected by screening and ‘c’ the number of cancers emerging after a negative test. The former is the ability of a test to detect a cancer and cannot be directly estimated since ‘c’ includes not only cancers missed by the test but also rapidly growing cancers not yet existing at the time of the test. Its estimation thus requires either modelling of the test reaction as a function of the presence of occult blood in the faeces, as calculated with the data of the Minnesota trial using a rehydrated test (Church et al, 1997), or modelling the MST (mean sojourn time) of the tumour as recently calculated with French data (Launoy et al, 1997). Due to this relatively low sensitivity of the test, the emergence of cancer among subjects with a negative test could become one of the problems physicians may face in mass screening. At present, data about such cancers is sparse.

The present study describes cancers emerging after a negative Haemoccult II® (without rehydration) from the data of the first round of screening in the Department of Calvados, and determines their incidence according to clinical parameters (sex, age, subsite and stage) and the time since the test. Using this incidence, the programme sensitivity, defined as the probability for an individual with detectable colorectal cancer to be detected by this programme, was estimated according to the same parameters.

*Received 7 July 1998*
*Revised 25 March 1999*
*Accepted 12 April 1999*

*Correspondence to:* V Bouvier

### POPULATION AND METHODS

Between April 1991 and the end of December 1994, a first round of screening for colorectal cancer with Haemoccult II® was progressively done in the six areas of the Department of Calvados (France). The population invited for screening comprised 165 000 people aged 45–74 years. The six areas were progressively included in the screening programme over 18 months. The tests were first proposed by general practitioners and occupational doctors. Letters were then sent out inviting people to obtain the test free of charge from their general practitioner or pharmacist. No dietary or drug restrictions were required. All tests were mailed to a single centre and were processed without rehydration. A test was considered positive when a blue colour appeared in the centre or diffused from the centre to the edges of the slide within 60 s after placing a drop of hydrogen peroxide in the centre. It was considered borderline when the blue stain was confined to the edges. If the result was positive or borderline, subjects were

**Table 1** Characteristics of colorectal cancer in Department of Calvados between 1991 and 1995 for people aged 45 to 74

| | Positive test | Negative test | Non-responders | Reference | Total |
|---|---|---|---|---|---|
| Sex | | | | | |
| Male | 94 (61.8) | 52 (52.0) | 207 (59.8) | 207 (58.1) | 560 |
| Female | 58 (36.2) | 48 (48.0) | 139 (40.2) | 149 (41.9) | 394 |
| Stage[a] | | | | | |
| I | 69 (45.4) | 28 (28.0) | 85 (24.6) | 84 (23.6) | 266 |
| II | 83 (54.6) | 72 (72.0) | 265 (75.4) | 272 (76.4) | 692 |
| Age | | | | | |
| 45–54 | 13 (8.5) | 10 (10.0) | 35 (10.1) | 35 (9.8) | 93 |
| 55–64 | 47 (31.0) | 35 (35.0) | 112 (32.4) | 120 (33.7) | 314 |
| 65–74 | 92 (60.5) | 55 (55.0) | 199 (57.5) | 201 (56.5) | 547 |
| Subsite | | | | | |
| Proximal | 26 (17.1) | 25 (25.0) | 78 (22.5) | 74 (20.8) | 203 |
| Distal | 98 (64.5) | 42 (42.0) | 155 (44.8) | 176 (49.4) | 471 |
| Rectum | 26 (17.1) | 33 (33.0) | 110 (31.8) | 106 (29.8) | 275 |
| Unknown | 2 (1.3) | 0 | 3 (0.9) | 0 | 5 |
| Total | 152 | 100 | 346 | 356 | 954 |

[a]Stage I: Dukes' A; Stage II: all the others.

**Table 2** Distribution of sex, age, stage and subsite of cancer occurring after a negative test according to the time since test

| | First year | Second year | Third year | Total |
|---|---|---|---|---|
| Sex | | | | |
| Male | 23 (51.1) | 16 (51.6) | 13 (54.2) | 52 |
| Female | 22 (48.9) | 15 (48.4) | 11 (45.8) | 48 |
| Stage[a] | | | | |
| I | 14 (31.1) | 10 (32.3) | 4 (16.7) | 28 |
| II | 31 (68.9) | 21 (67.7) | 20 (83.3) | 72 |
| Age | | | | |
| 45–54 | 4 (8.9) | 4 (12.9) | 2 (8.3) | 10 |
| 55–64 | 17 (37.8) | 10 (32.3) | 8 (33.3) | 35 |
| 65–74 | 24 (53.3) | 17 (54.8) | 14 (58.4) | 55 |
| Subsite | | | | |
| Proximal | 8 (17.8) | 11 (35.5) | 6 (25.0) | 25 |
| Distal | 24 (53.3) | 9 (29.0) | 9 (37.5) | 42 |
| Rectum | 13 (28.9) | 11 (35.5) | 9 (37.5) | 33 |
| Total | 45 | 31 | 24 | 100 |

[a]Stage I: Dukes' A; Stage II: all the others.

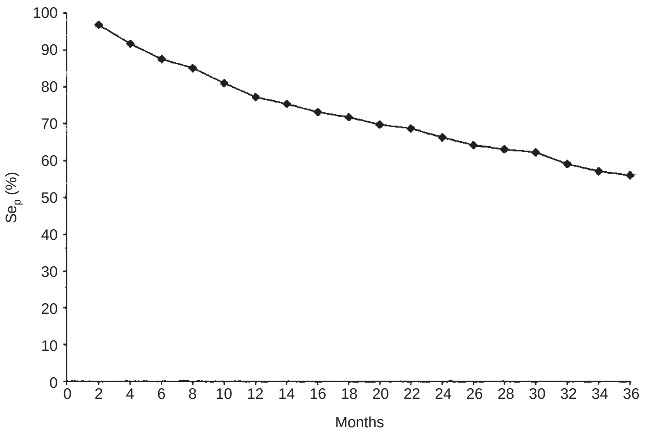

**Figure 1** Programme sensitivity according to time since test invited by their practitioner to undergo a colonoscopy. Screening organization and the test modality have been described in previous papers (Launoy et al, 1995, 1996). Of those invited for this first round of screening, 71 307 subjects completed the test (rate participation: 43.4%). The positivity rate was 2.8% (2020 positive tests). Among this population, 1603 (79.4%) were fully investigated (colonoscopy ± DCBE), and 1277 (63.2%) had a complete colonoscopy. Thus 152 cancers were diagnosed and the predictive positive value for cancer was 9.5%.

All the cancers diagnosed between 1991 and 1995 in people living in the department were recorded by the local digestive cancer registry, whether they occurred in a subject participating in the screening or not. In this way, four different groups were constituted:

1. Cancers occurring after a positive test in participating individuals (positive-test group)
2. cancers occurring after a negative test in participating individuals (negative-test group)
3. cancers occurring in people refusing to participate (refusers group)

 © 1999 Cancer Research Campaign

**Table 3** Incidence of colorectal cancer per 100 000 after a negative test according to time and clinical parameters

| Time | People at the beginning of the period | Cumulated incidence | Cumulated incidence according to sex | | Cumulated incidence according to age | | | Cumulated incidence according to subsite | | | Cumulated incidence according to stage[a] | |
|---|---|---|---|---|---|---|---|---|---|---|---|---|
| | | | Male | Female | 45–54 | 55–64 | 65–74 | Proximal | Distal | Rectal | I | II |
| 0–6 | 69 271 | 31.8 (18.5–45.0) | 34.3 | 29.9 | 4.1 | 37.2 | 62.9 | 4.3 | 23.1 | 7.2 | 11.5 | 26.0 |
| 7–12 | 69 249 | 65.0 (46.0–83.9) | 78.8 | 54.9 | 16.3 | 70.2 | 125.9 | 11.5 | 36.1 | 18.8 | 21.7 | 49.1 |
| 13–18 | 69 229 | 86.6 (64.7–108.6) | 102.8 | 74.9 | 24.4 | 90.9 | 167.9 | 21.7 | 43.3 | 23.1 | 27.4 | 65.0 |
| 19–24 | 62 702 | 111.4 (83.3–139.5) | 135.8 | 93.7 | 33.5 | 113.4 | 217.8 | 27.8 | 49.5 | 35.5 | 37.0 | 80.1 |
| 25–30 | 42 909 | 133.3 (89.5–177.2) | 173.9 | 103.5 | 43.8 | 129.6 | 262.0 | 33.8 | 57.6 | 39.4 | 42.7 | 96.3 |
| 31–36 | 26 648 | 173.1 (93.0–253.1) | 210.3 | 144.0 | 43.8 | 172.0 | 353.8 | 42.4 | 73.4 | 54.7 | 45.4 | 133.4 |

[a]Stage I: Dukes' A; Stage II: all the others.

4. cancers occurring before the offer of screening (reference group).

The follow-up was at least 12 months for all the negative test group, 24 months for 90.5% and 36 months only for 38.5% of it. These values were taken into account for the calculation of colorectal cancer incidence after a negative test. For example, people who completed the test in May 1994, with an 18-month follow-up, were considered as censored data over this period for the determination of incidence.

The programme sensitivity was estimated by $Se_p = a/a + c$, 'a' being the number of cancers detected with a positive test and 'c' the number of cancers occurring after a negative test. After 12 months, cancers emerging after a negative test were known only for people who had a long enough follow-up period. So after 12 months, 'c' in the formula $Se_p = a/a + c$ was estimated by applying an incidence calculated as above to the total number of negative tests.

Extension of cancers was classified according to two stages: stage I (Dukes'A: carcinoma not yet extended through the muscularis propria and no regional lymph node metastasis (Dukes, 1932)) and stage II for all the others. Subsite was classified according to three segments: the proximal colon including caecum, ascending colon, hepatic flexure and transverse colon; the distal colon with splenic flexure, descending colon, sigmoid colon and rectosigmoid, and the rectum.

The incidence and the programme sensitivity were calculated with Microsoft® Excel 5.0 software and for statistical analysis, SAS® System for Windows™, Release 6.10 software was used.

### RESULTS

### Cancers occurring after a negative test

From 1 January 1991 to 31 December 1995, 988 cancers were diagnosed in Calvados: 152 (16.0%) after a positive test (positive-test group), 100 (10.5%) after a negative test (negative-test group), 346 (36.3%) in the non-responder subjects (refusers group) and 356 (37.3%) before screening invitation (reference group). Thirty-four cancers were excluded: 22 (2.2%) for incomplete data and 12 (1.2%) cases diagnosed more than 36 months after a negative test.

Table 1 shows the distribution of clinical characteristics of cancer according to group. Cancers in the negative-test group were significantly different from those of the positive-test group, regarding stage ($P < 0.05$) and subsite ($P < 0.05$), but not from the refusers group or the reference group.

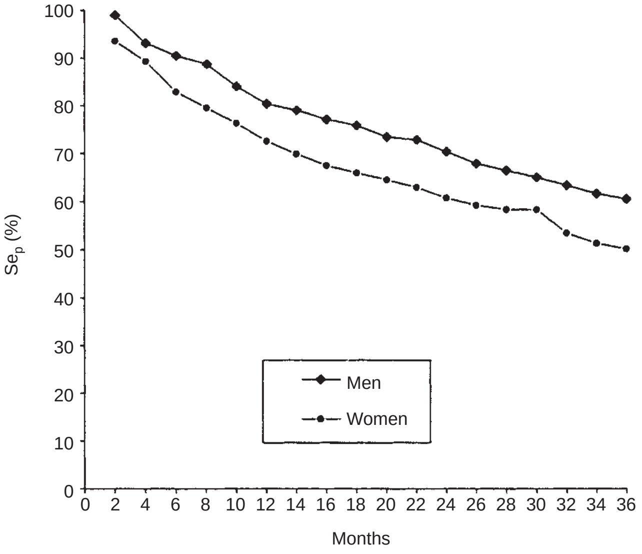

**Figure 2** Programme sensitivity according to sex.

Table 2 shows clinical characteristics (sex, age, stage, subsite) of cancers occurring after a negative test according to the time since test. No significant difference was found in distribution of sex, age, stage and subsite according to the time since test.

Table 3 shows the evolution of incidence of cancer among people with a negative test according to the clinical parameters. Mean incidence during this period was 57.7 per 100 000. In comparison, calculated incidence in the reference group for the same period was 141.6 per 100 000, more than twice the mean incidence in the negative-test group.

### Programme sensitivity after first round

Figures 1 and 2 show the evolution of sensitivity of the programme estimated as described above. Globally, programme sensitivity was 77.2% at 1 year, 66.3% at 2 years and 55.9% at 3 years.

Programme sensitivity followed the same evolution in time (Figure 2) for men and women. One year after the test it was respectively 80.3% and 72.5%; 70.3% and 60.7% after 2 years; and 60.5% and 50.1% 3 years after the test. The sensitivity ratio was quite stable with time for these 3 years (1.10, 1.15 and 1.20).

*© 1999 Cancer Research Campaign* 

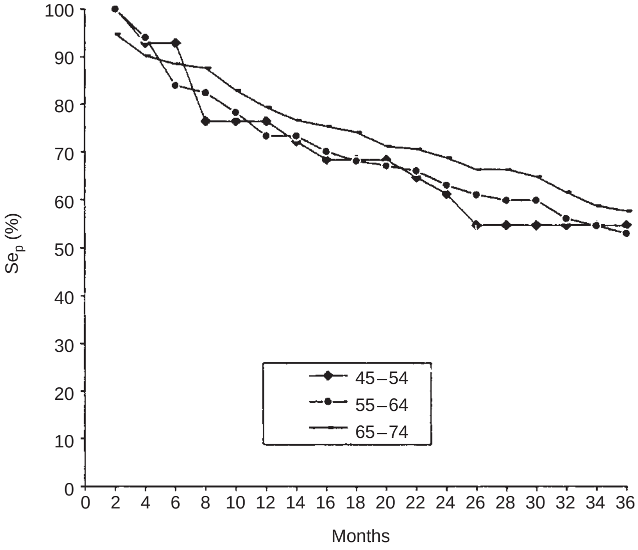

**Figure 3** Programme sensitivity according to age

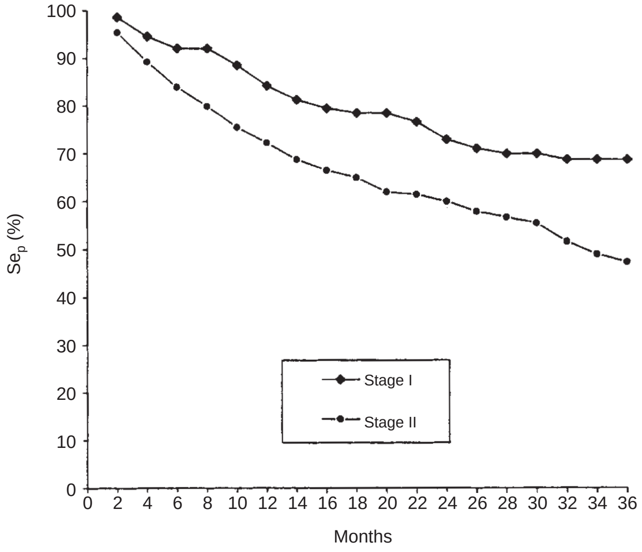

**Figure 5** Programme sensitivity according to stage

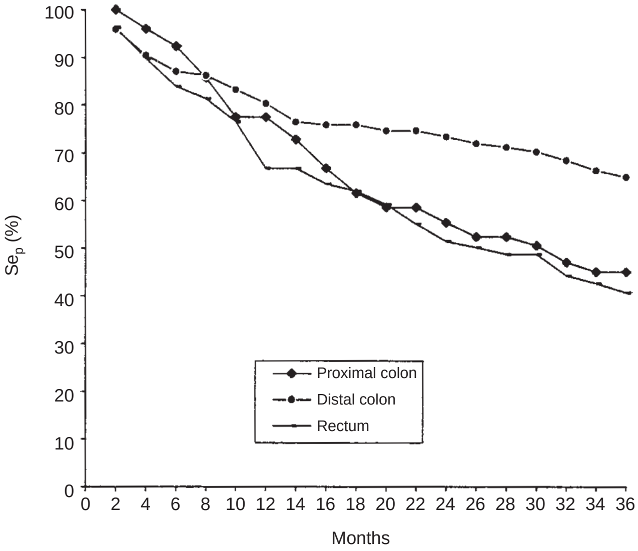

**Figure 4** Sensitivity of the programme according to the cancer subsite

Programme sensitivity was constantly better for people aged 65–74 years than for the others (Figure 3): 1 year after the test, the sensitivity was 79.3% (65–74 years) versus 73.4% (55–64 years) and 76.5% (45–54 years); the corresponding figures after 2 years were 68.9%, 63.1% and 61.3%, and after 3 years were 57.7%, 53.0% and 54.7%.

Figure 4 shows the evolution of programme sensitivity according to subsite. One year after the test, sensitivity was 80.3% for distal cancer, 77.4% for proximal cancer and 66.7% for rectal cancer. During the following period, sensitivity for distal colon was markedly different from the other two subsites. Two years after, programme sensitivity was 73.3% for distal cancer, 55.4% for proximal cancer and 51.4% for rectal cancer. Three years after, the corresponding figures were, respectively, 64.9, 45.0 and 40.7%. The ratio between distal cancer and other subsites increased in time: 1.15 after 1 years, 1.37 after 2 years and 1.51 after 3 years.

Programme sensitivity was better for less advanced cancer. One year after the test, it was 81.1% for stage I and 72.2% for stage II. The corresponding figures after 2 years were, respectively, 72.9% and 59.9%, and after 3 years, 68.7% and 47.3% (Figure 5).

### DISCUSSION

According to our results, cancers emerging after a negative test do not differ from those of the reference and non-responder groups, in particular for stage of extension. In the two European prospective trials, cancers emerging after a negative test were diagnosed with a better stage than those occurring in the control group (Hardcastle et al, 1996; Kronborg et al, 1996). This conflict could be due to a higher rate of Dukes' A stage among reference or non-responder subjects in our study (respectively 23.6% and 24.6%) than corresponding rates observed among the control groups in Fünen or Nottingham (11.0%), which revealed a difference in the health care systems of two European countries. The use of colonoscopy has been widespread in France since the 1980s, so access to colonoscopy is certainly easier in France. In fact, the percentage of stage I (Dukes'A) in our reference group is similar to that of the control group from Minnesota, where subjects were volunteers from a cancer society. In no study do cancers after a negative test present a worse extension than those of the reference group. Therefore, whatever the country, patients and physicians do not seem to be falsely reassured by a negative Haemoccult II® and are watchful of symptoms. In our study, the incidence in the negative-test group was about half that of the reference group, in accordance with the results of Allison showing that negative subjects had only half the likelihood of developing colorectal cancer than the general population (Allison et al, 1990).

From a public health point of view, programme sensitivity is of greater importance than test sensitivity, because it reflects programme efficacy after integrating several determinants such as test sensitivity and natural history of cancer. The best way to estimate programme sensitivity is to obtain available data from several

© 1999 Cancer Research Campaign rounds of screening. In our study, we estimated programme sensitivity after only one round. In this condition, our programme sensitivity was globally higher than that estimated in the other European trials. The general programme sensitivity was 77.2% 1 year after the test, while it was 50% in the study of Allison et al, and 89.3% in the Minnesota trial that used a rehydrated test. Two years after the test, it was 68.5%, which is higher than the calculated sensitivity from the Fünen (44.8–48.0%) and Nottingham (48.7–67.6%) trials. This difference may be due to the fact that our study focused only on the first round of screening, prevalent cancers detected with the test being more numerous for the first round than for the others. For example, using the data from Fünen, the sensitivity after the first round was 80.0% (37 detected cancers and nine interval cancers), whereas after two rounds the sensitivity fell to 55.0% (50 screen-detected cancers and 40 interval cancers) (Kronborg et al, 1989, 1996). It could also result from the difference in the positive rate of Haemoccult II®: 1–1.2% in Nottingham, 0.8–1.8% in Fünen, 1.4% in the Allison study and 2.8% in the Calvados programme. This variation of positive rates could be due to the dietary restriction 3 days before taking the test in Fünen, the repetition of testing after a first positive test with one to four positive slides in Nottingham and the consideration of a borderline test as positive in Calvados (Kronborg et al, 1987; Launoy et al, 1995; Robinson et al, 1995). Programme sensitivity was better for males and for subjects aged 65–74 years, in accordance with the results from the Minnesota and Fünen studies. Programme sensitivity was also different according to the subsite, and higher for the distal colon 1 year after the test, despite a higher incidence of distal cancer among the negative-test group in comparison with other subsites. This surface discrepancy may have two causes. First, distal cancers are the most frequent in the general population. For instance, between 1978 and 1990, crude incidence in Calvados was 35.9/100 000 for distal cancer, 26.2/100 000 for rectal cancer and 17.8/100 000 for proximal cancer (unpublished data). Secondly, regarding the natural history of colorectal cancer, the MST for distal cancer has been estimated to be about twice as long as the other two subsites: 6.44 years versus 3.49 years for proximal cancer and 2.61 years for rectal cancer (Launoy et al, 1997). It seems reasonable to think that the cancers emerging in the first year after a negative test are mainly missed cancers, and that the longer the time since the test, the higher the proportion of real surfacing cancers. Thus, since test sensitivity is similar for the various subsites (Launoy et al, 1997), programme sensitivity during the first year after the test is also similar. Moreover, since distal cancer grows more slowly than the others, programme sensitivity tends to be better for this localization in subsequent years.

The finding that cancers diagnosed after a negative Haemoccult II® do not have a worse stage of extension than those diagnosed among general population is an encouraging result, since it reduces the expected negative effect due to the low sensitivity of the test.

### ACKNOWLEDGEMENTS

We would like to thank Mrs Odile Gignoux Duron for her contribution to the editing of the final version.

### REFERENCES

Allison JE, Feldman R and Tekawa IS (1990) Hemoccult screening in detecting colorectal neoplasm: sensitivity, specificity and predicting value. *Ann Intern Med* **112**: 328–333

Benhamiche AM, Faivre J, Menegoz F and Grosclaude P (1997) Les cancers digestifs en France à l'aube de l'an 2000. *Hepato-Gastro* **4**: 8–12

Church TR, Ederer F and Mandel JS (1997) Faecal occult blood screening in the Minnesota study: sensitivity of the screening test. *J Natl Cancer Inst* **89**: 1440–1448

Dukes CE (1932) The classification of cancer of the rectum. *J Pathol Bacteriol* **35**: 323–332

Faivre J, Grosclaude P, Launoy G, Arveux P, Raverdy N, Menegoz F, Schaffer P, Daures JP and De Vathaire F (1997) Les cancers digestifs en France. Distribution géographique et estimation de l'incidence nationale. *Gastroentérol Clin Biol* **21**: 174–180

Hardcastle JD, Chamberlain JO, Robinson MH, Moss SM, Amar SS, Balfour TW, James PD and Mangham CM (1996) Randomised controlled trial of faecal-occult-blood screening for colorectal cancer. *Lancet* **348**: 1472–1477

Kronborg O, Fenger C, Sonderaard O, Pedersen KM and Olsen J (1987) Initial mass screening for colorectal cancer with faecal occult blood test. *Scand J Gastroenterol* **22**: 677–686

Kronborg O, Fenger C, Olsen J, Bech K and Sonderaard O (1989) Repeated screening for colorectal cancer with faecal occult blood test. A prospective randomised study at Funen, Denmark. *Scand J Gastroenterol* **24**: 599–606

Kronborg O, Fenger C, Olsen J, Jorgensen OD and Sondergaard O (1996) Randomised study of screening for colorectal cancer with faecal-occult-blood test. *Lancet* **348**: 1467–1471

Launoy G, Herbert C, Reaud JM, Thezee Y, Tichet J, Maurel J, Ollivier V, Pegulu L, Caces E, Valla A and Gignoux M (1995) Haemoccult test properties according to type and number of positive slides in mass screening for colorectal cancer. *Br J Cancer* **72**: 1043–1046

Launoy G, Herbert C, Vallee JP, Desoubeaux N, Reaud JM, Ollivier V, Bouvier V, Flachs A, Arsene D, Valla A and Gignoux M (1996) Le dépistage de masse du cancer colorectal en France. Expérience auprès de 165.000 personnes dans le Calvados. *Gastroentérol Clin Biol* **20**: 228–236

Launoy G, Smith TC, Duffy WS and Bouvier V (1997) Colorectal cancer mass-screening: estimation of faecal occult blood test sensitivity, taking into account cancer mean sojourn time. *Int J Cancer* **73**: 220–224

Mandel JS, Bond JH, Church TR, Snover DC, Bradely MB, Schuman LM and Ederer F (1993) Reducing mortality from colorectal cancer by screening for faecal occult blood. *N Engl J Med* **328**: 1365–1371

Robinson MH, Moss SM, Hardcastle JD, Whynes DK, Chamberlain J and Mangham CM (1995) Effect of retesting with dietary restriction in Haemoccult screening for colorectal cancer. *J Med Screen* **2**: 41–44.

© 1999 Cancer Research Campaign 
